# Testing the Risk of Child Hyperactivity-Inattention Problems in Families Living with Housing-cost Burden during the COVID-19 Pandemic

**DOI:** 10.1007/s10802-025-01413-y

**Published:** 2026-01-16

**Authors:** Jun-Hong Chen, Cao Fang, Jesse J. Helton, Michael G. Vaughn, Yuanyuan Yang

**Affiliations:** 1 School of Social Work, Saint Louis University, Tegeler Hall, 3550 Lindell Blvd, St. Louis, MO 63103 USA; 2 George Warren Brown School of Social Work, Washington University in St. Louis, 1 Brookings Dr, St. Louis, MO 63130 USA; 3 Anne and Henry Zarrow School of Social Work, The University of Oklahoma, 700 Elm Ave, Norman, OK 73069 USA

**Keywords:** Child hyperactivity-inattention, Housing burden, The COVID-19 pandemic, Generalized propensity score weighting

## Abstract

Housing cost burden is a critical issue in contemporary society, not only because of the economic strain it imposes on households, but also due to its impact on child development outcomes. This study examines the risk of child hyperactivity-inattention problems across different levels of housing cost burden during the COVID-19 pandemic and explores whether these patterns differ between renter and homeowner families, two groups that experienced distinct challenges to child well-being during this period. Addressing this research gap is crucial for identifying children at high risk of hyperactivity-inattention problems, particularly during times of severe socioeconomic adversity. Using secondary data from the Panel Study of Income Dynamics (*N* = 2,589), we applied negative binomial regression models supported by generalized propensity score weighting to strengthen causal inference. The results indicate that among homeowner families, higher housing cost burden is not significantly associated with an increased risk of child hyperactivity-inattention problems. In contrast, among renter families, the risk does not rise significantly until housing costs consume more than 30% of total income. These findings provide timely evidence in support of policies that promote housing affordability as a strategy to safeguard child development, a particularly pressing concern as policy debates consider cuts to social assistance programs addressing housing and child support needs for families most at risk.

## Introduction

Child hyperactivity-inattention problems constitute a significant health concern due to their profound effects on social functioning and emotional regulation, which elevate the risk for depression, substance use, and antisocial behavior (Giannotta & Rydell, 2016; Lorenzo et al., 2021). Empirical evidence has established that the prevalence of child hyperactivity-inattention problems increases in contexts of family economic stress, such as income instability or financial hardship (Choi et al., 2017; Keilow et al., 2020). During the COVID-19 pandemic, housing burden, shown as a high ratio of housing costs to household income, emerged as a salient form of economic stress (Malpezzi, 2023). Although prior studies have linked economic stress to elevated risk of hyperactivity-inattention symptoms in children (Choi et al., 2017; Keilow et al., 2020), it remains unclear whether similar associations apply to housing burden specifically, and how these associations may differ by homeownership status. Addressing this gap is essential for a better understanding of which populations face the most elevated risks of hyperactivity-inattention problems during periods of severe socioeconomic adversities.

### The Relationship between Household Economic Stress and Child Hyperactivity-Inattention Problems

From the perspective of the Family Stress Model theoretical framework, child hyperactivity-inattention problems are more prevalent in economic stress contexts due to the cascading effects of economic hardship on family functioning (Totsika et al., 2020). The model posits that financial strain, such as income shortage, leads to increased parental stress, anxiety, and depressive symptoms, which in turn impair the quality of parenting. In such conditions, parents may become less emotionally available, more irritable, or inconsistent in discipline, creating a chaotic or unstructured home environment (Masarik & Conger, 2017). This instability can disrupt a child’s emotional and behavioral development, increasing the likelihood of difficulties with self-regulation, attention, and impulse control, which are key features of hyperactivity-inattention problems (Totsika et al., 2020). In addition to the Family Stress Model framework, acknowledging the unique context of the COVID-19 pandemic could further strengthen the rationale explaining the elevated risk of child hyperactivity-inattention problems. During the pandemic, parents spent substantially more time with their children, including in academic contexts while managing virtual schooling (Calear et al., 2022). This increased proximity and involvement may have heightened parents’ awareness or perception of their children’s attentional and activity-level difficulties, a risk factor turning into stress that affects child behavioral development (Pickren et al., 2022). Notably, for socioeconomically disadvantaged families, parents were more vulnerable to face stress during the COVID-19 pandemic (Loose et al., 2023).

Neurobiological evidence also supports the association between household economic stress and increased risks of child hyperactivity-inattention problems. Exposure to stressors such as financial instability can dysregulate children’s hypothalamic-pituitary-adrenal axis, leading to elevated cortisol levels that affect neural connectivity and plasticity (Doom et al., 2018; Sripada et al., 2014). These neurobiological changes may impair cognitive control and emotional regulation, increasing vulnerability to symptoms of inattention and hyperactivity (Smith & Pollak, 2020). Together, these findings suggest that household economic stress is a risk factor exposing children to the risk of hyperactivity-inattention problems.

### Housing Cost Burden: an Important Index of Household Economic Stress

Housing burden refers to the percentage of a household’s income that is spent on housing costs, including rent or mortgage payments, utilities, and other related expenses. A common standard used by policymakers and researchers, established by the U.S. Department of Housing and Urban Development, defines a household as housing burdened if it spends more than 30% of income on housing and severely housing cost burdened if this ratio exceeds 50%. These definitions are also widely used in reports by the Congressional Research Service (Daniels et al., 2025). This metric is a critical indicator of economic stress because it reflects how much of a household’s financial resources are consumed by basic shelter needs, leaving less available for other essentials (Acolin & Reina, 2022). High housing burden can signal financial instability, increase the risk of eviction or foreclosure, and contribute to cycles of poverty, making it a key measure in assessing housing affordability and overall economic well-being.

During the COVID-19 pandemic, housing burden intensified due to widespread economic disruptions and income loss. Specifically, people faced job losses, reduced work hours, or unstable employment, sharply decreasing their ability to afford rent or mortgage payments (Ringlein et al., 2024). At the same time, housing costs in many areas remained stable or even increased, widening the gap between income and housing expenses (Arcaya et al., 2020). This mismatch between income shortage and housing costs pushed households, especially low-income renters, into housing cost burden or severe burden status (Malpezzi, 2023). As such, these households faced increased risks of eviction, overcrowding, and homelessness, underscoring the critical role of housing affordability in determining economic resilience and its influences on one’s health (Mehdipanah, 2020).

### Homeownership: a Potential Divide for Child Outcomes in Low-Income Contexts

During economic stress events, such as the COVID-19 pandemic, homeownership status is a key facilitator in maintaining child well-being for a family (Rahman & Steeb, 2024). Homeownership plays an important role in providing a sense of stability and control over one’s living environment (Desmond & Wilmers, 2019). Such a secure environment is a known protective factor for child development (Leventhal & Newman, 2010). In contrast, renters, especially during periods of economic instability, were more likely to experience residential instability, such as the threat of eviction. These challenges can increase parental stress, disrupt children’s routines, and lead to negative behavioral outcomes such as heightened emotional and behavioral problems (Hanson, 2025; Leventhal & Newman, 2010). This view aligns with Grinstein-Weiss et al. (2010), who find that when under financial pressure, homeowners are more likely than renters to continuously maintain structured routines for their children, an essential element of positive development. On the other hand, both renter and homeowner families could have similar experience that housing costs overwhelm their income, which can, in turn, negatively impact child development (Masarik & Conger, 2017). Despite these insights, it is unclear whether there are differences in the risk of hyperactivity–inattention problems between children in homeowner and renter households.

### The Current Study

It has been well established that children in low-income contexts are at greater risk for developing behavioral and emotional problems (Masarik & Conger, 2017). However, it is unclear whether the risk of child hyperactivity-inattention problems is higher in families living with more severe housing burden and whether such patterns could vary by homeownership, an important but often overlooked dimension of child well-being during the pandemic (Rahman & Steeb, 2024). To bridge these research gaps, this study investigated the risk of child hyperactivity-inattention problems across different housing burden levels during the COVID-19 pandemic among renter families and homeowner families. Addressing this gap is crucial to enhance our capacity to better identify children having high risk of hyperactivity-inattention problems, especially during times of severe socioeconomic adversities.

## Methods

### Data

This study draws on data collected from children and their families from the Panel Study of Income Dynamics (PSID), incorporating both the Main Study and the Child Development Supplement (CDS). Conducted annually from 1968 to 1997 and biennially thereafter, the PSID data for this study are drawn from the 2019 and 2021 wave, thereby ensuring the temporal order by reflecting 2020 household economic information and 2021 child outcomes while including history of child emotional and behavioral problems in the pre-COVID period as a covariate to account pre-existing difference. Specifically, the PSID Main Study provides comprehensive information on household economic well-being, including household income, wealth, and housing expenditures, as well as detailed parental demographic characteristics such as age, gender, race, education level, and employment status at baseline (2019). The Child Development Supplement (CDS) complements these data by including validated measures of parental psychological distress and children’s hyperactivity–inattention problems. To maintain temporal ordering, which ensures that all covariates precede the measurement of children’s behavioral outcomes, measures of parenting behaviors and parental psychological distress at baseline (2019) were used in the analyses.

By combining information from both the Main Study and the CDS, the dataset offers a robust foundation for the analyses conducted in this study. For a complete picture showing how child hyperactivity-inattention problems vary by housing-cost burden, the study focuses on families who participated in both the PSID Main Study and the CDS. The final analytic sample includes 2,589 children, with an average age of 7.9 years (SD = 4.3) and 50.3% were identified as female. This study did not require Institutional Review Board (IRB) approval or informed consent because the analyses used de-identified, publicly available secondary data and involved no identifiable human subjects.

## Measures

### Child Hyperactivity–Inattention Problems

Child hyperactivity–inattention problems were reported by parents and assessed as a continuous variable using the Hyperactivity–Inattention subscale of the Strengths and Difficulties Questionnaire (SDQ; Goodman et al., 1998). The SDQ is a widely validated behavioral screening tool designed to assess children’s emotional and behavioral functioning. The hyperactivity–inattention subscale consists of five items that capture children’s levels of restlessness, fidgeting, distractibility, impulsivity, and difficulty sustaining attention. Each item is rated on a 3-point scale (0 = not true, 1 = somewhat true, 2 = certainly true), and the total subscale score ranges from 0 to 10, with higher scores indicating greater levels of hyperactivity and inattention symptoms. In this study, the continuous total score was used to capture variation in children’s behavioral regulation across the sample. Internal consistency was acceptable, with Cronbach’s alpha exceeding 0.80. Factor analysis supported the unidimensional structure of the construct, with only one factor exhibiting an eigenvalue greater than 1, suggesting that the items reflect a single underlying dimension of hyperactivity-inattention problems.

### Housing Cost Burden

 In this study, housing cost burden is treated as a categorical variable, measured by the ratio of total housing expenditure to total household income adjusted for family size. Following the definition by the Congressional Research Service (Daniels et al., 2025), this ratio is used to classify households into three categories: low housing-cost burden (housing costs less than 30% of income), housing burdened (housing costs between 30% and 50% of income), and severely housing burdened (housing costs exceeding 50% of income). Housing expenditure, based on data from the PSID, includes key components such as mortgage or rent payments, property taxes, homeowner’s insurance, and utility costs (e.g., electricity, gas, water). These components collectively reflect the financial demands of maintaining residence needs and are used to assess the extent to which housing expenses strain household income.

Covariates include parent, child, and family demographic characteristics. Parent characteristics included age (in years), gender (male or female), employment status (employed or unemployed), educational attainment (college degree or higher), and psychological distress.

### Parent Psychological Distress

Parent psychological distress is measured as a continuous variable derived from the Kessler Psychological Distress Scale (K6) included in the PSID. The K6 is a widely validated screening instrument designed to assess psychological distress consisting of six items that ask respondents how frequently they have felt nervous, hopeless, restless or fidgety, so depressed that nothing could cheer them up, that everything was an effort, and worthless. Each item is rated on a 5-point Likert scale ranging from 0 = none of the time to 4 = all of the time, and the total score reflects the overall level of psychological distress, with higher scores indicating greater distress. In this study, the total K6 score was treated as a continuous measure to capture the full variability of parental psychological distress (Kessler et al., 2002). Higher scores reflect greater psychological distress.

Regarding race, it includes non-Hispanic White American, non-Hispanic Black American, Hispanic, Asian, Native American, Pacific Islander, multiracial, or any other racial group. To enable multivariate analyses, race is collapsed into non-Hispanic White, non-Hispanic Black, and Other.

Child characteristics comprised age (in years), gender (male or female), and a binary indicator of whether the child had ever consulted a doctor or health professional for a problematic behavioral issue.

Household characteristics included parenting structure (dual-parenting or single-parenting) and household wealth. Warm and punitive parenting behaviors are measured using items from the PSID Child Development Supplement that assessed the frequency of parents’ engagement in positive and harsh parenting practices. Warm parenting reflects the frequency with which parents display affectionate and supportive behaviors toward their children, such as showing warmth, praise, and physical affection. Punitive parenting captures the frequency of disciplinary or harsh behaviors, such as scolding or spanking. Parents reported how often they engaged in each behavior during a typical week, allowing these measures to capture the weekly frequency of both positive and punitive interactions. Higher scores on the warm parenting scale indicate more frequent expressions of warmth and affection, whereas higher scores on the punitive parenting scale indicate more frequent use of harsh disciplinary practices.

Household income is measured as the ratio of total family income to the U.S. federal official poverty threshold, which is adjusted for family size. Total family income includes both earnings and social benefits. Household wealth is measured as the ratio of the household wealth to the U.S. federal official poverty threshold, which is adjusted for family size. Based on data set availability, household wealth includes savings and other assets minus its debts. Savings included saving and checking accounts. Other assets include farm or business, bonds, vehicles, and financial investments (e.g., stocks).

### Analytic Approaches

To address the overdispersion characteristic of the Poisson-distributed outcome variable, this study employed negative binomial regression models to examine how the risk of child hyperactivity-inattention problems varies by housing-cost burden levels. To strengthen causal inference and account for potential selection bias across different levels of housing-cost burden, generalized propensity score weighting was applied. As a quasi-experimental method, generalized propensity score weighting was employed to ensure observations across different housing-cost burden levels share similar demographic characteristics, thereby improving the comparability of observations (Guo & Fraser, 2014). Additionally, the history of child behavioral problems was included as a covariate to adjust for pre-existing differences in the outcome, allowing for a more accurate comparison of child hyperactivity-inattention problems risk across housing-cost burden levels.

Following Guo and Fraser (2014), to create a generalized propensity score weight, a multinomial logistic regression model was first run to predict the probability of each housing-cost burden level, ***P***_***n***_, where ***n*** represents the number of housing-cost burden levels. Demographic characteristics were included as independent variables to predict probability ***P***_***n***_. These demographic characteristics include parent age, gender, race, employment status, educational attainment, parent psychological distress, child age, gender, history of child behavioral problems, family structures, warm and punitive parenting, and household income, and household wealth. Next, we utilized the predicted probability ***P***_***n***_ to create a generalized propensity score by the inverse probability, namely ***1/P***_***n***_. By ensuring demographic characteristics and history of child behavioral problems to be similar across different housing-cost burden levels (i.e., similar demographic features across low housing burden, housing burdened, and severe housing burden), the application of generalized propensity score weight enabled us to better compare child hyperactivity-inattention problems between different housing-cost burden levels.

To further mitigate estimation bias and strengthen causal inference, this study employed a doubly robust estimation approach to disentangle the effects of housing-cost burden from confounding influences such as demographic characteristics and prior child behavioral problems (Funk et al., 2011; Tan, 2010). Doubly robust estimation is a method in causal analysis that integrates two modeling strategies, namely outcome regression and treatment assignment modeling, offering consistent effect estimates. Specifically, the first component estimated the outcome as a function of covariates to adjust for confounders, while the second model estimated the probability of treatment (i.e., different levels of housing-cost burden) conditional on the same covariates. By combining these models, the approach enhanced estimation robustness and allows for more accurate identification of treatment effects. This dual adjustment ensured that differences in the risk of child hyperactivity-inattention problems were more plausibly attributed to variations in housing-cost burden levels.

Overall, the analytic approaches based on the combination of generalized propensity score weight and doubly robust estimation were utilized for the following purposes: In simple terms, when comparing the risk of child hyperactivity-inattention problems between low, medium, and high housing cost burden populations, a generalized propensity score weight was used to ensure all covariates are “similar”, i.e., non-significant difference. By doing so, when all covariates are similar for all people, we can be more confident that the differences observed in children’s hyperactivity–inattention problems are mainly due to differences in housing cost burden, rather than other underlying factors. Regarding the incident risk ratio, values higher than one indicate higher risk of child hyperactivity-inattention problem.

The analyses were conducted separately for homeowner families (*n* = 1,393) and renter families (*n* = 1,196). Although this study also tested an interaction model, which included the interaction between housing-cost burden and homeownership and yields similar results, we report findings based on a subgroup approach. This strategy compared child hyperactivity-inattention problems across different housing-cost burden levels within each subgroup categorized by homeownership status. Such an approach helped avoid bias that can arise when drawing conclusions about subpopulations from aggregate data (Yang et al., 2021). Therefore, while an interaction model provided similar results, this study presented primary results based on the strategy where comparison of child hyperactivity-inattention problems between different housing-cost burden levels were analyzed separately for homeowners and renters. Results of an interaction model were presented as a supplement in Appendix Table 6.

## Results

Table 1 highlights key demographic differences between homeowners and renters. Homeowners tended to be older and employed. Homeowners also experience lower psychological distress. Renters were more likely to be members of minority groups and in lower income brackets. Household income and wealth during the COVID-19 pandemic was higher among homeowners compared to renter families. Compared to homeowner families, a greater proportion of renter families experienced housing-cost burden. Child hyperactivity-inattention problems were higher among renter families compared to homeowner families.

**Table 1:** Descriptive results (*N* = 2,589)

	Homeowner Families (*n* = 1,393)	Renter Families (*n* = 1,196)	*p*-value
**Parent characteristics**			
Age	37.8 (SD = 7.6)	34.3 (SD = 7.7)	*P* < 0.001
Female	76.6%	82.2%	*P* < 0.001
Race			*P* < 0.001
White American	68.9%	33.8%	
Black American	23.9%	57.6%	
Others	7.2%	8.6%	
Employed	78.2%	67.8%	*P* < 0.001
College or above	53.8%	16.9%	*P* < 0.001
Psychological distress	3.5 (SD = 3.4)	4.8 (SD = 4.2)	*P* < 0.001
**Child characteristics**			
Age	7.9 (SD = 4.3)	7.8 (SD = 4.2)	*P* = 0.506
Female	51.3%	49.0%	*P* = 0.237
History of behavioral problem	17.1%	17.0%	*P* = 0.948
Child hyperactivity-inattention problems	2.8 (SD = 2.5)	3.1 (SD = 2.5)	*P* = 0.008
**Family characteristics**			
Single-parent family	16.7%	51.5%	*P* < 0.001
Warm parenting	13.6 (SD = 11.7)	11.5 (SD = 11.4)	*P* < 0.001
Punitive parenting	2.2 (SD = 4.0)	1.6 (SD = 6.0)	*P* = 0.019
Household income-to-poverty ratio	5.1 (SD = 7.4)	2.0 (SD = 1.7)	*P* < 0.001
Household wealth-to-poverty ratio	3.3 (SD = 8.5)	0.5 (SD = 3.4)	*P* < 0.001
Housing burden			*P* < 0.001
Housing-to-income ratio < 0.3	68.7%	32.6%	
0.3 ≤ Housing-to-income ratio < 0.5	21.4%	27.6%	
Housing-to-income ratio ≥ 0.5	9.9%	39.8%	

Table 2 showed, ***among renter families*** (*n* = 1,196), the risk of child hyperactivity-inattention problems significantly varied by household income during the COVID-19 pandemic. Specifically, among renter families, compared to children in families experiencing low housing-cost burden (housing-to-income ratio < 30%), the risk of child hyperactivity-inattention problems was significantly higher among children in families experiencing housing burdened (30% ≤housing-to-income ratio < 50%) (aIRR = 1.25, 95% CI: 1.07–1.47, *p* = 0.004) and families experiencing severe housing burdened (housing-to-income ratio ≥ 50%) (aIRR = 1.24, 95% CI: 1.07–1.45, *p* = 0.006). Table 3 showed, ***among homeowner families*** (*n* = 1,393), the risk of child hyperactivity-inattention problems did not significantly rise as housing-cost burden increases during the COVID-19 pandemic. Noticeably, results shown in the table of Appendix Table 6 showed that the risk of child hyperactivity-inattention problems in the reference category (i.e., to children in families experiencing low housing-cost burden) were similar for both homeowner and renter families. These findings further support our findings by excluding the concern that results difference between children in homeowner and renter families were due to disparities in the reference group. Also, sensitivity analyses using a more nuanced categories (housing-to-income ratio lower than 25%, between 25% and 50%, between 50% and 75%, and above 75%) showed similar results presented in the table of Appendix Table 7.

**Table 2:** Negative binomial regression model applied with generalized propensity score weight ***among renter families*** (Outcome: child hyperactivity-inattention problems)

	Incident rate ratio	*p*-value	95% C.I.
**Main predictor**			
Low housing burden: housing-to-income ratio < 0.3 (ref)			
Housing burdened: 0.3 ≤ housing-to-income ratio < 0.5	1.25	0.004	[1.07, 1.47]
Severe housing burdened: housing-to-income ratio ≥ 0.5	1.24	0.006	[1.07, 1.45]
**Parent covariates**			
Age	0.99	0.042	[0.98, 1.00]
Female	0.85	0.085	[0.71, 1.02]
Race			
White American (ref)			
Black American	0.89	0.127	[0.77, 1.03]
Others	0.94	0.483	[0.79, 1.12]
Employed	0.88	0.075	[0.76, 1.01]
College or above	0.96	0.608	[0.81, 1.13]
Psychological distress	1.02	0.005	[1.01, 1.03]
**Child covariates**			
Age	0.95	< 0.001	[0.93, 0.96]
Female	0.88	0.037	[0.79, 0.99]
History of behavioral problem	2.46	< 0.001	[2.17, 2.79]
**Family covariates**			
Single-parent family	0.94	0.347	[0.82, 1.07]
Warm parenting	1.00	0.539	[1.00, 1.01]
Punitive parenting	1.02	0.003	[1.01, 1.03]
Household income	0.84	0.044	[0.70, 0.99]
Household wealth	0.87	0.028	[0.77, 0.98]

Incident rate ratio lower than 1 indicates lower risk of experiencing the outcome

**Table 3:** Negative binomial regression model applied with generalized propensity score weight ***among Homeowner families*** (Outcome: child hyperactivity-inattention problems)

	Incident rate ratio	*p*-value	95% C.I.
**Main predictor**			
Low housing burden: housing-to-income ratio < 0.3 (ref)			
Housing burdened: 0.3 ≤ housing-to-income ratio < 0.5	0.90	0.504	[0.67, 1.22]
Severe housing burdened: housing-to-income ratio ≥ 0.5	1.00	0.993	[0.63, 1.58]
**Parent covariates**			
Age	0.98	0.044	[0.96, 1.00]
Female	2.04	0.036	[1.05, 3.96]
Race			
White American (ref)			
Black American	0.98	0.908	[0.73, 1.32]
Others	1.04	0.865	[0.66, 1.63]
Employed	1.29	0.106	[0.95, 1.75]
College or above	1.10	0.686	[0.70, 1.72]
Psychological distress	1.08	< 0.001	[1.04, 1.13]
**Child covariates**			
Age	0.97	0.197	[0.92, 1.02]
Female	1.01	0.960	[0.68, 1.49]
History of behavioral problem	2.95	< 0.001	[2.29, 3.80]
**Family covariates**			
Single-parent family	1.13	0.337	[0.88, 1.43]
Warm parenting	0.98	0.012	[0.97, 1.00]
Punitive parenting	1.09	< 0.001	[1.05, 1.14]
Household income	0.97	0.458	[0.89, 1.06]
Household wealth	0.92	< 0.001	[0.89, 0.95]

Incident rate ratio lower than 1 indicates lower risk of experiencing the outcome

Tables 4 and 5 showed the results of a balance test in renter and homeowner families, respectively. Specifically, the findings showed that demographic characteristics did not significantly differ across different levels of housing cost burden. These demographic characteristics included parent age, gender, race and ethnicity, employment status, educational attainment, parent psychological distress, child age, gender, history of child behavioral problems, family structures, parenting, and household income and wealth. By ensuring these demographic characteristics to be similar across different housing-cost burden levels, the application of generalized propensity score weight allowed a valid comparison of child hyperactivity-inattention problems across different housing burden levels in renter and homeowner families.

**Table 4:** Balance test of generalized propensity score weighting: covariate comparison across different housing-to-income levels among ***Renter families***

	*P*-value before weighting	*P*-value after weighting
**Parent covariates**		
Age	*P* = 0.007	*P* = 0.757
Female	*P* < 0.001	*P* = 0.813
Race	*P* < 0.001	*P* = 0.890
Employment status	*P* = 0.002	*P* = 0.992
Education	*P* < 0.001	*P* = 0.935
Psychological distress	*P* = 0.437	*P* = 0.623
**Child covariates**		
Age	*P* = 0.440	*P* = 0.385
Female	*P* = 0.347	*P* = 0.750
Behavioral problem history	*P* = 0.779	*P* = 0.769
**Family covariates**		
Single-parent family	*P* < 0.001	*P* = 0.858
Warm parenting	*P* = 0.022	*P* = 0.839
Punitive parenting	*P* = 0.956	*P* = 0.877
Household income	*P* < 0.001	*P* = 0.807
Household wealth	*P* = 0.105	*P* = 0.967

1. After applying generalized propensity score weighting, people in different housing burden groups share similar baseline demographic features (i.e., no significant difference in baseline demographic features across different housing-to-income levels)

2. By ensuring these people in different housing-to-income levels share similar baseline demographic features, such a balance test justifies the use of generalized propensity score weighting to get a valid causal inference of comparing child hyperactivity-inattention problem across different housing-to-income levels

**Table 5:** Balance test of generalized propensity score weighting: covariate comparison across different housing-to-income levels among ***Homeowner families***

	*P*-value before weighting	*P*-value after weighting
**Parent covariates**		
Age	*P* = 0.083	*P* = 0.898
Female	*P* = 0.130	*P* = 0.287
Race	*P* < 0.001	*P* = 0.060
Employment status	*P* = 0.064	*P* = 0.072
Education	*P* < 0.001	*P* = 0.364
Psychological distress	*P* = 0.770	*P* = 0.315
**Child covariates**		
Age	*P* = 0.173	*P* = 0.332
Female	*P* = 0.554	*P* = 0.252
Behavioral problem history	*P* = 0.809	*P* = 0.758
**Family covariates**		
Single-parent family	*P* < 0.001	*P* = 0.137
Warm parenting	*P* = 0.212	*P* = 0.360
Punitive parenting	*P* = 0.135	*P* = 0.638
Household income	*P* < 0.001	*P* = 0.203
Household wealth	*P* = 0.284	*P* = 0.763

1. After applying generalized propensity score weighting, people in different housing burden groups share similar baseline demographic features (i.e., no significant difference in baseline demographic features across different housing-to-income levels)

2. By ensuring these people in different housing-to-income levels share similar baseline demographic features, such a balance test justifies the use of generalized propensity score weighting to get a valid causal inference of comparing child hyperactivity-inattention problem across different housing-to-income levels

## Discussion

The findings of this study revealed that the risk of child hyperactivity and inattention varies across levels of housing cost burden and differs by homeownership status. Specifically, for renter families, reducing housing costs to 30% or less of household income appeared to be a protective factor for children’s behavioral outcomes during periods of severe socioeconomic adversity. On the other hand, for homeowner families, the risk of hyperactivity-inattention problems did not significantly increase even when housing costs exceed 50% of income. These results do not diminish the importance of housing affordability for homeowners but suggest that homeownership may provide a buffering context against the negative effects of economic stress on child behavior, especially during economic downturns. Importantly, our findings offer timely evidence supporting policies that promote housing affordability as a strategy to safeguard child development, particularly important as the current policies consider cutting social assistance that address housing and child support needs among families in greatest need in the United States.

This study also shows that both a child’s history of behavioral problems and parental psychological distress are strong predictors of hyperactivity-inattention symptoms across both renter and homeowner families. Early behavioral difficulties in children, such as impulsivity or emotional dysregulation, often set the stage for ongoing attention-related challenges (Chen, 2024). Parental psychological distress, including depression, anxiety, or chronic stress, can undermine parenting capacity and increase the emotional strain within the household, further exacerbating child behavioral issues (Gulenc et al., 2018; Marchetti et al., 2020). Additionally, the findings suggest that attention to punitive parenting practices may be important, given their observed associations with the risk of child hyperactivity–inattention problems among both renter and homeowner families. Together, these findings highlight the critical need for early and family-based interventions that target both the child’s behavioral needs and parental mental health across diverse housing and socioeconomic contexts.

Notably, among homeowner families, this study found a higher risk of child hyperactivity–inattention problems in female-led households. It could suggest that more parenting or child care support is more in need in female-led households than male-led households. To provide a clearer picture, this study includes a sensitivity analysis that considered interaction effects by caregiver gender and race. Results (Appendix Table 8) indicate that the main findings, which show higher risk of child hyperactivity-inattention problems in renter families under greater housing cost burden, remain consistent after accounting for potential interaction effects simultaneously brought by gender and race.

The findings of this study underscore the importance of considering housing burden as a critical factor when identifying children at heightened risk for hyperactivity-inattention problems, particularly during periods of severe socioeconomic adversity. Families facing economic stress are at high risk of experiencing elevated levels of psychological distress that undermine the caregiving environment (Neppl et al., 2016). These cascading effects can compromise parenting consistency and emotional responsiveness, exacerbating children’s emotional and behavioral problems (Chen et al., 2024). Therefore, screening for risk of child behavioral problems in the practical settings should integrate housing and broader socioeconomic factors to more accurately target children who may benefit from behavioral interventions.

Our findings highlight the critical role of homeownership and reduced housing cost burdens in supporting better behavioral outcomes for children. However, achieving homeownership and alleviating housing-related financial strain remain significant challenges, particularly for economically disadvantaged populations. Structural barriers, including a persistent shortage of affordable housing, continue to limit the feasibility of homeownership (Acolin et al., 2019; Desmond & Wilmers, 2019). Compounding these issues are inadequate public investment, limited rental assistance programs, and the slow development of affordable housing options, all of which hinder efforts to reduce housing cost burdens (Davidson, 2016; Reid, 2023). These interrelated factors underscore the complexity of addressing housing affordability and securing housing access. To support wealth accumulation among low-income populations and improve pathways to homeownership, it is essential to narrow the gap between income and basic living expenses (McKernan, 2008). This study therefore recommends that the current policies discontinue social assistance cut in a way that would widen the income-needs gap for families in greatest needs.

It is important to recognize that attentional regulation and hyperactivity represent related but partially distinct dimensions of neurobehavioral functioning, rather than a fully unidimensional construct (Marcus & Barry, 2011; Smith et al., 2013). Empirical and neurocognitive research suggests that while these domains frequently co-occur, they are underpinned by both shared and unique mechanisms involving executive control, arousal regulation, and inhibitory processes (Salum et al., 2014). Consequently, the phenotypic spectrum of hyperactivity–inattention symptoms is continuous and heterogeneous, encompassing normative variability in attentional control as well as clinically significant impairments consistent with Attention-Deficit/Hyperactivity Disorder (Chaulagain et al., 2023). In the current study, child hyperactivity–inattention problems were assessed using the Strengths and Difficulties Questionnaire (SDQ) available in the PSID. Although the SDQ offers a reliable dimensional indicator of behavioral regulation difficulties, it does not differentiate between symptom domains or clinical thresholds. As such, our findings should be interpreted as capturing general variation in attentional and hyperactivity-related behaviors, rather than diagnostic classifications. Future studies employing comprehensive diagnostic tools or multi-informant clinical assessments are warranted to replicate and refine these associations.

To the best of our knowledge, no single theoretical model has been specifically developed to explain the association between economic stress and hyperactivity–inattention while systematically excluding other behavioral domains. Existing frameworks, such as the family stress model framework, provide valuable insights into how economic hardship influences child outcomes via parental stress and disrupted parenting, but they generally apply to a broad range of behavioral, emotional, and social problems rather than isolating attentional and hyperactive behaviors (Masarik & Conger, 2017). Further theoretical development is therefore needed to identify mechanisms that selectively account for the effects of economic stress on hyperactivity–inattention. Advancing such models would improve the specificity of research and inform interventions targeting economic stress–related attentional and self-regulation difficulties in children.

This study has several limitations. As there is no perfect dataset, certain important factors, such as genetic information, which may significantly influence child behavioral outcomes, were not included. However, to address potential pre-existing differences, the study employs generalized propensity score weighting, ensuring that baseline behavioral histories among children are not statistically different from each other. For future research that incorporates genetic or biological variables, this study provides a valuable benchmark for comparison, enabling deeper investigation into whether and how the risk of child hyperactivity and inattention varies under high housing cost burdens. Notably, the relative costs and accessibility of renting versus home ownership could differ between rural and urban areas, with urban regions typically facing higher housing prices and more competitive rental markets, while rural areas may have lower costs but limited availability and fewer amenities (Díaz-Dapena et al., 2024). Additionally, the housing data in the PSID comprise multiple components that differ between renters and homeowners (e.g., inclusion of insurance costs, property taxes, or maintenance expenses for owners versus rent payments for tenants). These structural differences may limit direct comparability between the two groups. However, given the nature of the secondary data used in this study, we do not conduct relevant analyses. More efforts are required to clarify whether findings of this study could vary by these factors. Despite its limitations, the study underscores that homeownership could be a protective factor for a lower risk of child hyperactivity-inattention problems. Similarly, reducing housing cost burdens to below 30% of household income could offer similar protective effects, particularly during periods of severe socioeconomic adversity. These findings contribute important insights into how overlapping economic disadvantages shape child behavioral outcomes in challenging times.

## Conclusion

Living in family economic stress contexts during the COVID-19 pandemic is a risk factor for child development. Our findings suggest that homeownership may serve as a protective context for a lower risk of child hyperactivity-inattention problems in families experiencing high housing cost burden during economic downturns. For renter families, our findings suggest that reducing housing cost burdens to below 30% of household income could offer similar protective effects. These results highlight the importance of incorporating housing cost burden into proactive screening to identify children at elevated risk for hyperactivity-inattention issues during times of socioeconomic hardship. Importantly, our findings offer timely evidence supporting policies that promote housing affordability as a strategy to safeguard child development, especially important as current policy debates consider cuts to social assistance programs that address housing and child support needs for families with the greatest need.

## Data Availability

The authors do not have permission to share data.

## Appendix

**Table 6:** Marginal effects of the interaction model (*N*=2,589)

Interaction between homeownership and housing-cost burden	Incidence Count	*P*-value of comparison
**Housing-cost burden among homeowner families**		
***Low housing burden***: housing-to-income ratio < 0.3	2.69	
***Housing burdened***: 0.3 ≤ housing-to-income ratio < 0.5	2.82	(Housing burden) - (Low housing burden): 0.482
***Severe housing burdened***: housing-to-income ratio ≥ 0.5	2.62	(Severe housing burden) - (Low housing burden): 0.781
**Housing-cost burden among renter families**		
***Low housing burden***: housing-to-income ratio < 0.3	2.68	
***Housing burdened***: 0.3 ≤ housing-to-income ratio < 0.5	3.23	(Housing burden) - (Low housing burden): 0.009
***Severe housing burdened***: housing-to-income ratio ≥ 0.5	3.12	(Severe housing burden) - (Low housing burden): 0.024

No statistically significant difference in the low housing burden between homeowner and renter families (*p*>0.05)

All results are adjusted by socioeconomic demographic characteristics (i.e., covariates shown in the Tables 2 and 3)

**Table 7:** Sensitivity analyses

	Incident rate ratio	*p*-value	95% C.I.
**Hosing burden among renter families**			
housing-to-income ratio < 0.25 (ref)			
0.25 ≤ housing-to-income ratio < 0.50	1.21	0.007	[1.05, 1.39]
0.50 ≤ housing-to-income ratio < 0.75	1.29	0.004	[1.08, 1.53]
housing-to-income ratio ≥ 0.75	1.24	0.029	[1.02, 1.50]
**Hosing burden among homeowner families**			
housing-to-income ratio < 0.25 (ref)			
0.25 ≤ housing-to-income ratio < 0.50	1.02	0.691	[0.91, 1.15]
0.50 ≤ housing-to-income ratio < 0.75	0.98	0.826	[0.78, 1.22]
housing-to-income ratio ≥ 0.75	0.85	0.366	[0.59, 1.22]

All results are adjusted by socioeconomic demographic characteristics (i.e., covariates shown in the Tables 2 and 3)

**Table 8:** Sensitivity analyses testing potential inequalities in gender and race

	Incident rate ratio	
	Homeowners	Renters
**Main effect**		
**Housing cost burden**		
Low housing burden (ref)		
Housing burdened	0.98	1.15
Severe housing burdened	0.74	1.78***
**Gender**		
Male (ref)		
Female	0.92	1.00
**Race**		
White American (ref)		
Black American	0.97	1.07
Others	0.89	0.87
**Two-way interaction effect**		
**Housing cost burden x Gender**		
Housing burdened x Female	0.93	1.09
Severe housing burdened x Female	1.32	0.93
**Housing cost burden x Race**		
Housing burdened x Black American	0.81	0.87
Housing burdened x Others	1.34	0.94
Severe housing burdened x Black American	0.98	0.79
Severe housing burdened x Others	0.95	1.73
**Gender x Race**		
Female x Black American	1.06	0.81
Female x burdened x Others	0.71	1.29
**Three-way interaction effect**		
**Housing cost burden x Gender x Race**		
Housing burdened x Female x Black American	1.15	1.23
Housing burdened x Female x Others	0.84	0.74
Severe housing burdened x Female x Black American	0.89	1.48
Severe housing burdened x Female x Others	1.01	1.20

[1]. Covariates shown in Tables 2 and 3 are also held for control for these sensitivity analyses

[2]. An example of Interpretation of the interaction effect: For “Severe housing burdened x Female”, it suggests that the differences between severe burden and low burden (ref) among ***Female parents*** are not significantly different from the differences between severe burden and low burden (ref) among ***Male parents*** (ref)
